# Efficacy and safety of airway clearance techniques in Chinese adult patients with pneumonia: a systematic review and meta-analysis

**DOI:** 10.3389/fmed.2026.1819629

**Published:** 2026-07-22

**Authors:** Hongyun Cai, Lijing Wang, Wei Yuan, Ying Qi

**Affiliations:** 1 Department of Cardiovascular, Affiliated Hospital of Hebei University, Lianchi District, Baoding City, Hebei Province, China; 2 Department of Neurology, Affiliated Hospital of Hebei University, Lianchi District, Baoding City, Hebei Province, China; 3 Department of Respiratory and Critical Care Medicine, Affiliated Hospital of Hebei University, Lianchi District, Baoding City, Hebei Province, China; 4 Department of Tuberculosis, Affiliated Hospital of Hebei University, Lianchi District, Baoding City, Hebei Province, China

**Keywords:** critical care, physical therapy modalities, pneumonia, respiratory therapy, systematic review

## Abstract

**Objective:**

This systematic review and meta-analysis aimed to synthesize the available evidence from Chinese randomized controlled trials to evaluate the efficacy and safety of airway clearance techniques compared to conventional treatment in adult patients with pneumonia, including stroke-associated pneumonia, severe viral pneumonia, and severe pneumonia requiring mechanical ventilation.

**Methods:**

We systematically searched PubMed, Web of Science, Cochrane Library, CNKI, and Wanfang databases from inception to January 1, 2026, for randomized controlled trials (RCTs) comparing airway clearance techniques plus conventional treatment *vs*. conventional treatment alone. Two reviewers independently conducted study selection, data extraction, and quality assessment. Data were pooled using a random-effects model in Stata, with between-study heterogeneity evaluated.

**Results:**

Eleven randomized controlled trials were included. Meta-analysis showed airway clearance techniques significantly increased treatment effectiveness (OR 3.83, 95% CI 2.04–7.16; I^2^ = 0%) and reduced complications (OR 0.16, 95% CI 0.07–0.41; I^2^ = 0%). They also improved arterial oxygen saturation (SMD 1.71, 95% CI 1.24–2.19), albeit with considerable heterogeneity (I^2^ = 76.2%). No significant effect was found on the mMRC Dyspnea Score (SMD−3.64, 95% CI −7.45–0.18), with very high heterogeneity (I^2^ = 98.1%).

**Conclusion:**

In conclusion, this meta-analysis provides hypothesis-generating evidence that airway clearance techniques may improve treatment effectiveness, reduce complications, and enhance oxygenation in Chinese adult patients with pneumonia. However, due to heterogeneity, methodological limitations, and lack of long-term patient-centered outcomes, these findings require confirmation in future international multicenter trials.

## 1 Introduction

Pneumonia is a common infectious disease with substantial global morbidity and mortality, posing a particularly significant burden on the elderly and patients with underlying comorbidities (1, 2). For adults with pneumonia requiring hospitalization, effective airway secretion management is a critical component of clinical care, as it impacts gas exchange, infection control, and ultimate clinical outcomes (3). In clinical practice, specialized airway clearance techniques are frequently employed as adjunctive therapies alongside conventional treatment (including oxygen therapy and pharmacotherapy) with the aim of improving prognosis (4–6).

However, conclusions regarding the precise efficacy and safety of these airway clearance techniques for adult patients with pneumonia are inconsistent across existing clinical studies, and there is a lack of high-quality synthesized evidence. Several recent randomized controlled trials conducted in China have evaluated the effects of various airway clearance strategies (e.g., evidence-based refined airway management, positioning management, fiberoptic bronchoscopy-assisted clearance) combined with conventional treatment. These studies primarily reported outcome measures including treatment effective rate, complication rate, arterial oxygen saturation, and the modified British Medical Research Council Dyspnea Score. Currently, there is no systematic review and meta-analysis specifically focusing on airway clearance techniques for adult pneumonia patients that is based on this body of recent Chinese trial data.

Therefore, this systematic review and meta-analysis aims to rigorously synthesize the current best evidence from Chinese randomized controlled trials to comprehensively evaluate the efficacy and safety of airway clearance techniques for adult patients with pneumonia, specifically in terms of treatment effective rate, complication rate, improvement in oxygen saturation, and dyspnea relief.

## 2 Method

This systematic review was retrospectively registered on INPLASY (registration number: INPLASY202650150). However, an internal protocol was developed *a priori*, and the review is reported in strict accordance with the Preferred Reporting Items for Systematic Reviews and Meta-Analyses (PRISMA) statement (7) (Supplementary Table 1).

### 2.1 Search strategy

We employed a systematic three-step search process to identify all relevant studies. Searches were conducted in the following electronic databases: PubMed, Web of Science, Cochrane Library, China National Knowledge Infrastructure (CNKI), and Wanfang Data. The search timeframe was set from the inception of each database until January 1, 2026. The search strategy combined Medical Subject Headings (MeSH) terms and free-text words to encompass studies related to adult pneumonia and various airway clearance techniques. The detailed search strings are provided in Supplementary Table 2. All searches were limited to published randomized controlled trials, with language restrictions to Chinese or English. Two investigators independently performed the searches across all databases.

### 2.2 Inclusion and exclusion criteria

#### 2.2.1 Types of studies

We included randomized controlled trials (RCTs) published in Chinese or English before January 1, 2026 that compared different airway management strategies.

#### 2.2.2 Types of participants

The study population consisted of adult hospitalized patients aged >=18 years with a clinical or radiographic diagnosis of pneumonia (including community-acquired and hospital-acquired pneumonia).

#### 2.2.3 Interventions

The intervention in the experimental group consisted of one or more active airway clearance techniques (e.g., evidence-based refined airway management protocols, positioning strategies, chest wall vibration, or specific breathing techniques) combined with conventional treatment (including but not limited to oxygen therapy, pharmacotherapy, routine nebulization and humidification, fiberoptic bronchoscopy, or high-flow humidified oxygen therapy). The control group received the same conventional treatment but without the aforementioned targeted active airway clearance techniques.

#### 2.2.4 Outcome measures

Included studies had to report at least one of the following primary outcome measures: treatment effective rate (Supplementary Table 3), complication rate, arterial oxygen saturation, or the modified British Medical Research Council Dyspnea Score.

#### 2.2.5 Exclusion criteria

We excluded studies with non-RCT designs, those involving pediatric or adolescent populations, interventions that incorporated early mobilization or general exercise training (to avoid confounding effects on outcomes), and studies from which relevant outcome data could not be extracted.

### 2.3 Assessment of methodological quality

Two investigators independently assessed the methodological quality and risk of bias of all eligible full-text articles using the Cochrane Collaboration's Risk of Bias Tool 1.0 (RoB1). This tool contains seven core domains for RCT evaluation: random sequence generation (selection bias), allocation concealment (selection bias), blinding of participants and personnel (performance bias), blinding of outcome assessment (detection bias), incomplete outcome data (attrition bias), selective reporting (reporting bias), and other potential sources of bias. Each domain was graded as low risk, unclear risk, or high risk of bias. Discrepancies during assessment were resolved via group discussion or consultation with a third senior reviewer. Overall risk of bias was defined as follows: overall low risk if all seven domains were rated low risk; overall high risk if any single domain was rated high risk; overall some concerns if no high-risk domains existed but one or more domains were rated unclear risk. RevMan 5.4 software was used to generate risk of bias summary plots.

### 2.4 Data extraction

Data extraction was performed independently by two investigators using a pre-designed, standardized form. The extracted information included study characteristics (e.g., first author, publication year, study design, country, sample size), participant demographics and clinical characteristics (e.g., age, sex, type of pneumonia), detailed descriptions of the intervention and control measures, and all relevant outcome data. For continuous variables (e.g., arterial oxygen saturation), we extracted the mean, standard deviation, and sample size for each group; for dichotomous variables (e.g., treatment effective rate, complication rate), we extracted the number of events and the total sample size for each group. If outcomes were reported at multiple time points within a study, data from the post-intervention assessment or the study-defined primary endpoint were extracted. Following independent extraction, the two investigators cross-checked their data sheets, and any discrepancies were resolved by re-examining the original article or through discussion. If data were missing or unclear in the published report, we attempted to contact the corresponding authors via email to obtain the necessary information. If no response was received, the data were marked as unavailable.

### 2.5 Statistical methods

For dichotomous outcomes (treatment effective rate, complication rate), the odds ratio was used as the effect measure. For continuous outcomes (arterial oxygen saturation, mMRC Dyspnea Score), the standardized mean difference was used as the effect measure. All meta-analyses were performed using the random-effects model for data pooling. Heterogeneity between studies was assessed using the I^2^ statistic (expressed as a percentage) and Cochrane's Q test (with a significance level set at *p* < 0.10). We interpreted it using the following thresholds: 0% to 40% might not be important; 30% to 50% may represent moderate heterogeneity; 50% to 75% may represent substantial heterogeneity; and 75% to 100% represents considerable heterogeneity. Meta-analyses were conducted using Stata software (version 18.0) to generate forest plots and pooled effect estimates with their 95% confidence intervals. Sensitivity analysis was performed by iteratively removing each individual study to observe the changes in the pooled effect estimate and its confidence interval. Assessment for publication bias was planned only for outcomes with 10 or more included studies (which was not the case for any outcome in this review). Risk of bias assessment for the included studies was performed using RevMan software (version 5.4) in accordance with the Cochrane Collaboration's RoB1 tool. The certainty of evidence for each outcome was assessed using the Grading of Recommendations Assessment, Development and Evaluation (GRADE) approach. A detailed GRADE summary is presented in Supplementary Table 4.

## 3 Results

### 3.1 Study selection

**Figure 1:**
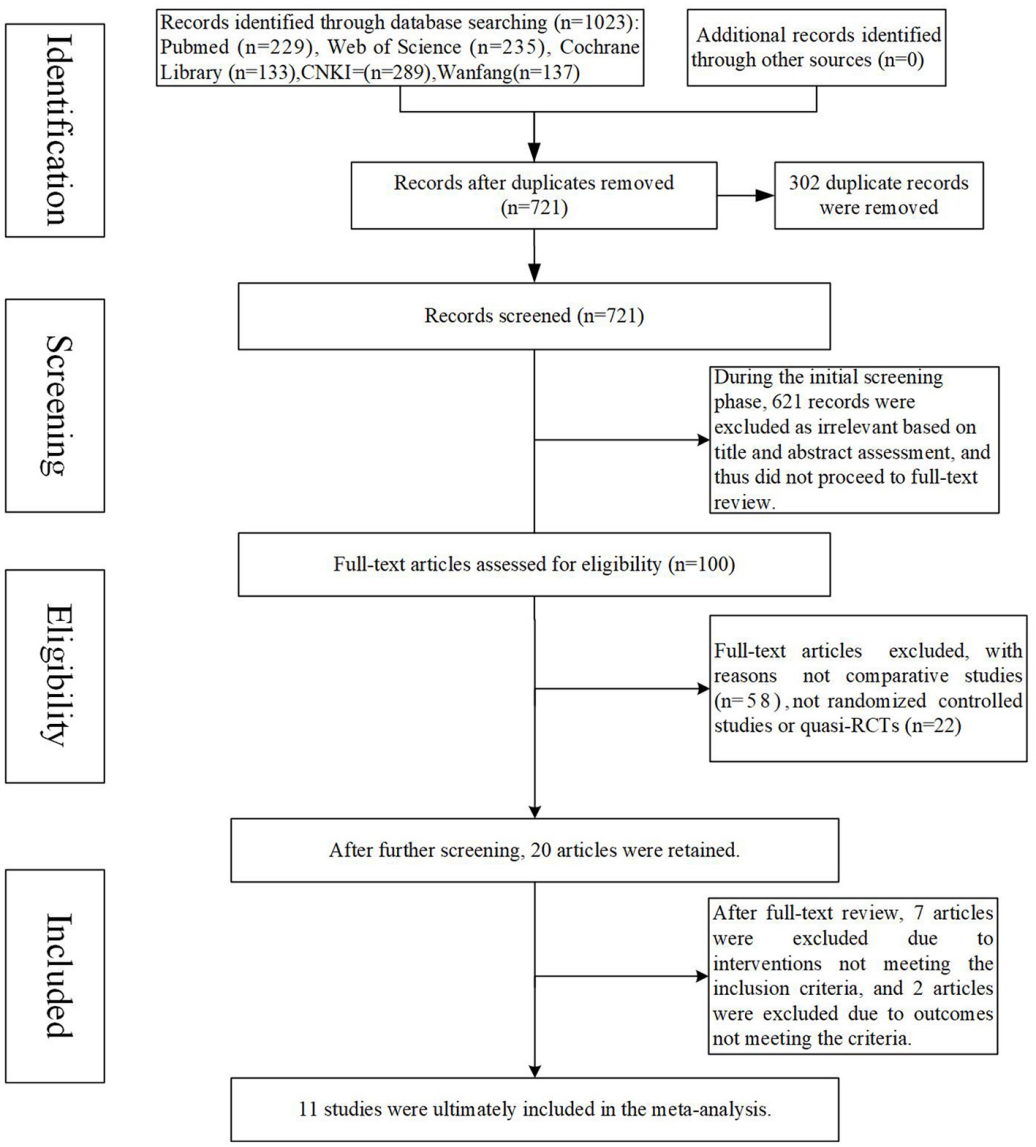
Flow diagram of study selection.

A total of 1,023 records were identified through database searching: 229 from PubMed, 235 from Web of Science, 133 from Cochrane Library, 289 from CNKI, and 137 from Wanfang. No additional records were identified through other sources. After removing 302 duplicate records, 721 unique records remained for screening. Based on title and abstract assessment, 621 irrelevant records were excluded during the initial screening phase. Subsequently, 100 full-text articles were assessed for eligibility. During the full-text review, 89 articles were excluded: 58 for being non-comparative studies, 22 for not being RCTs or quasi-RCTs, 7 for interventions not meeting the inclusion criteria, and 2 for outcomes not meeting the criteria. Among the excluded full-text articles, no randomized controlled trials conducted outside China met the inclusion criteria. The primary reasons for exclusion of non-Chinese studies were: (1) the intervention did not consist of active airway clearance techniques as defined in our protocol (e.g., studies focused solely on early mobilization or exercise); (2) the outcome measures did not include treatment effective rate, complication rate, arterial oxygen saturation, or the mMRC Dyspnea Score; or (3) the study population was not restricted to adult patients with pneumonia (e.g., mixed ICU populations without pneumonia-specific subgroup data). Ultimately, 11 studies were included in the systematic review and subsequent meta-analysis. All screening and exclusion numbers are consistent with the staged data presented in the PRISMA flow diagram (Figure 1).

### 3.2 Characteristics of included studies

A total of 11 randomized controlled trials were included in this systematic review, with their basic characteristics detailed in Table 1 (8–18). All studies were conducted in China, with sample sizes ranging from 40 to 200 participants. The mean age of participants varied widely across studies, ranging from approximately 45 to 76 years. The included populations comprised stroke-associated pneumonia, stable severe viral pneumonia, elderly severe pneumonia, general adult community/hospital-acquired pneumonia, and severe pneumonia requiring mechanical ventilation. The interventions primarily involved combining specific active airway clearance techniques or refined airway management protocols with conventional treatment (which included routine symptomatic treatment, nebulization and humidification, high-flow humidified oxygen therapy, or fiberoptic bronchoscopy). The control groups received the same conventional treatment but without the adjunctive active airway clearance techniques. Regarding the reported outcome measures: 7 studies reported treatment effective rate; 4 studies reported arterial oxygen saturation; 3 studies reported complication rate; and 3 studies reported the modified British Medical Research Council Dyspnea Score.

**Table 1:** General characteristics of the included RCTs.

Included study	Study design	Region	Sample size	Male (T/C)	Age (T/C) [Mean ±SD]	Pneumonia type/severity	Interventions (T/C)	Outcome indicators
Chen et al. (8)	RCT	China	40	NR	NR	Stroke-associated pneumonia	T: Routine nebulization and humidification + airway clearance techniques C: Routine nebulization and humidification	mMRC dyspnea score
Zhang et al. (9) (Shandong)	RCT	China	200	T: 52, C: 54	T: 50.53 **±** 7.70, C: 50.45 **±** 7.63	General adult community/hospital-acquired pneumonia	T: Evidence-based refined airway management + airway clearance techniques C: Routine care	Arterial oxygen saturation (SaO2), complication rate
Zhang et al. (10) (Xianyang)	RCT	China	60	T: 17, C: 16	T: 49.62 **±** 8.15, C: 50.62 **±** 8.21	General adult community/hospital-acquired pneumonia	T: Evidence-based detailed airway nursing + airway clearance techniques C: Routine care	Arterial oxygen saturation (SaO2), complication rate
Cao et al. (11)	RCT	China	100	T: 31, C: 29	T: 68.54 **±** 4.67, C: 69.02 **±** 4.53	Stroke-associated pneumonia	T: Routine treatment + airway clearance techniques C: Routine treatment	Treatment effective rate, mMRC Dyspnea Score
Zibinuer et al. (12)	RCT	China	50	T: 15, C: 17	T: 75.88 **±** 10.04, C: 72.00 **±** 10.09	Stable severe viral pneumonia	T: Routine treatment + airway clearance techniques + positioning management C: Routine treatment	Arterial oxygen saturation (SpO2)
Mo et al. (13)	RCT	China	60	T: 18, C: 19	T: 57.87 **±** 16.55, C: 59.27 **±** 15.22	Stroke-associated pneumonia	T: Routine treatment + airway clearance techniques C: Routine treatment + traditional postural drainage	Treatment effective rate
Qingling (14)	RCT	China	60	T: 20, C: 21	T: 72.05 **±** 3.06, C: 72.09 **±** 3.01	Elderly severe pneumonia	T: Airway clearance techniques C: Routine nebulization and humidification	Treatment effective rate, mMRC Dyspnea Score
Wang et al. (15)	RCT	China	50	T: 15, C: 16	T: 62.20 **±** 9.28, C: 60.80 **±** 9.12	Stroke-associated pneumonia after tracheotomy	T: High-flow humidified oxygen therapy + airway clearance C: High-flow humidified oxygen therapy	Treatment effective rate
Lu et al. (16)	RCT	China	200	T: 55, C: 54	T: 55.65 **±** 7.45, C: 55.45 **±** 7.25	Severe pneumonia with mechanical ventilation	T: Fiberoptic bronchoscopy + airway clearance therapy C: Fiberoptic bronchoscopy	Treatment effective rate
Lin et al. (17)	RCT	China	80	T: 21, C: 22	T: 45.8 **±** 15.8, C: 45.2 **±** 15.7	Severe pneumonia with mechanical ventilation	T: Routine treatment + fiberoptic bronchoscopy + airway clearance therapy C: Routine treatment	Treatment effective rate
Ma et al. (18)	RCT	China	150	T: 40, C: 39	T: 58.25 **±** 6.25, C: 57.98 **±** 6.33	Severe pneumonia with mechanical ventilation	T: Conventional symptomatic treatment + fiberoptic bronchoscopy + airway clearance therapy C: Conventional symptomatic treatment	Treatment effective rate, arterial oxygen saturation (SaO2), complication rate

NR, Not reported. Chen et al. (8) did not report detailed gender and age data of enrolled participants in the published full text.

### 3.3 Bias risk assessment

The methodological quality and risk of bias of all included randomized controlled trials (RCTs) were assessed using the Cochrane Collaboration's RoB1 tool. Seven domains were evaluated for each study: random sequence generation (selection bias), allocation concealment (selection bias), blinding of participants and personnel (performance bias), blinding of outcome assessment (detection bias), incomplete outcome data (attrition bias), selective reporting (reporting bias), and other potential sources of bias. Each domain was classified as having a low risk of bias, an unclear risk of bias, or a high risk of bias.

**Figure 2:**
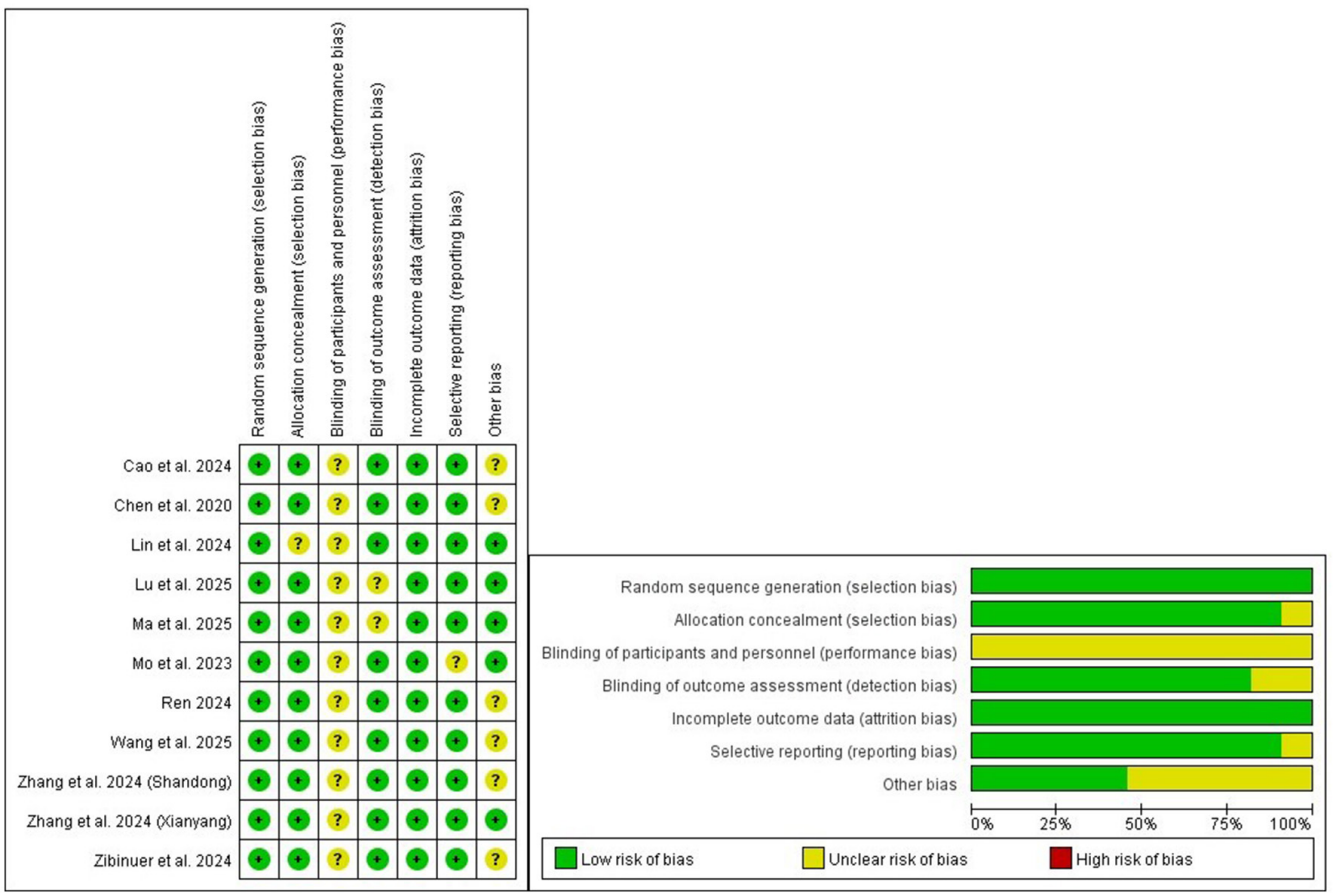
Summary proportion plot of risk of bias in included studies.

The risk of bias summary and distribution across studies are presented in Figure 2. All included studies were judged to have a low risk of bias in the domains of random sequence generation and incomplete outcome data. For blinding of participants and personnel (performance bias), all studies were rated as having some concerns (unclear risk) because the physical nature of airway clearance techniques precludes true blinding of participants or treating staff. No included study was judged to have a high risk of bias in any domain (Figure 2).

### 3.4 Meta-analysis of treatment effective rate

**Figure 3:**
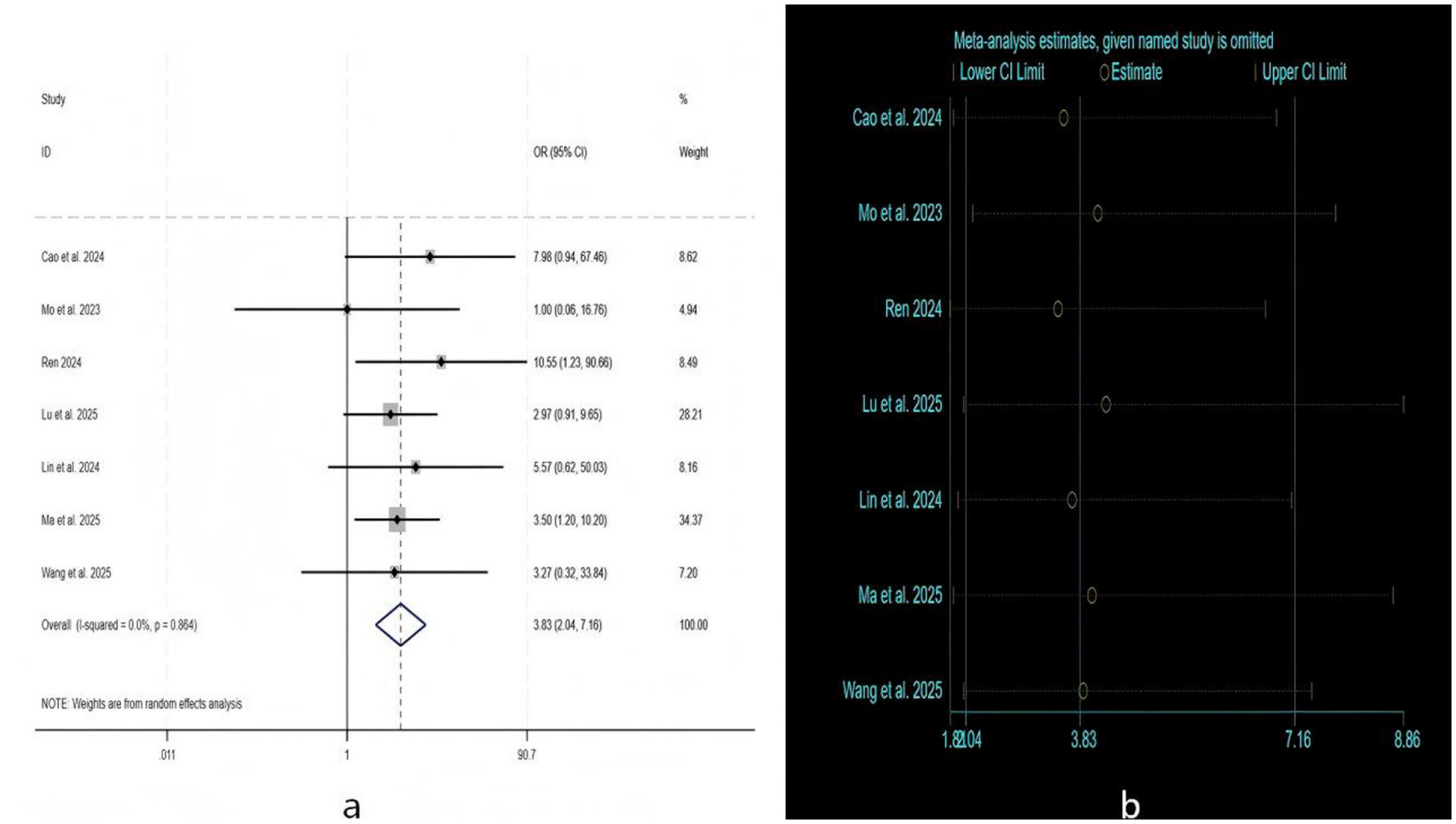
Meta-analysis results for treatment effective rate; **(a)** forest plot; **(b)** sensitivity analysis.

Seven studies reported the treatment effective rate and were included in the meta-analysis. A random-effects model was employed for the pooled analysis. The forest plot (Figure 3a) indicated no significant statistical heterogeneity among the studies (I^2^ = 0.0%, *p* = 0.864). The pooled analysis demonstrated that patients receiving airway clearance interventions had a significantly higher likelihood of treatment effectiveness compared to the control group, with a pooled odds ratio of 3.83 (95% CI: 2.04–7.16). Sensitivity analysis was performed by iteratively removing each individual study (Figure 3b). The results showed that neither the point estimate of the pooled effect size nor the lower and upper limits of its 95% confidence interval changed substantially after the removal of any single study, indicating that the findings of the meta-analysis regarding treatment effective rate are robust. The operational definitions of treatment effective rate for each of the seven contributing studies are provided in Supplementary Table 3.

### 3.5 Meta-analysis of complication rate

**Figure 4:**
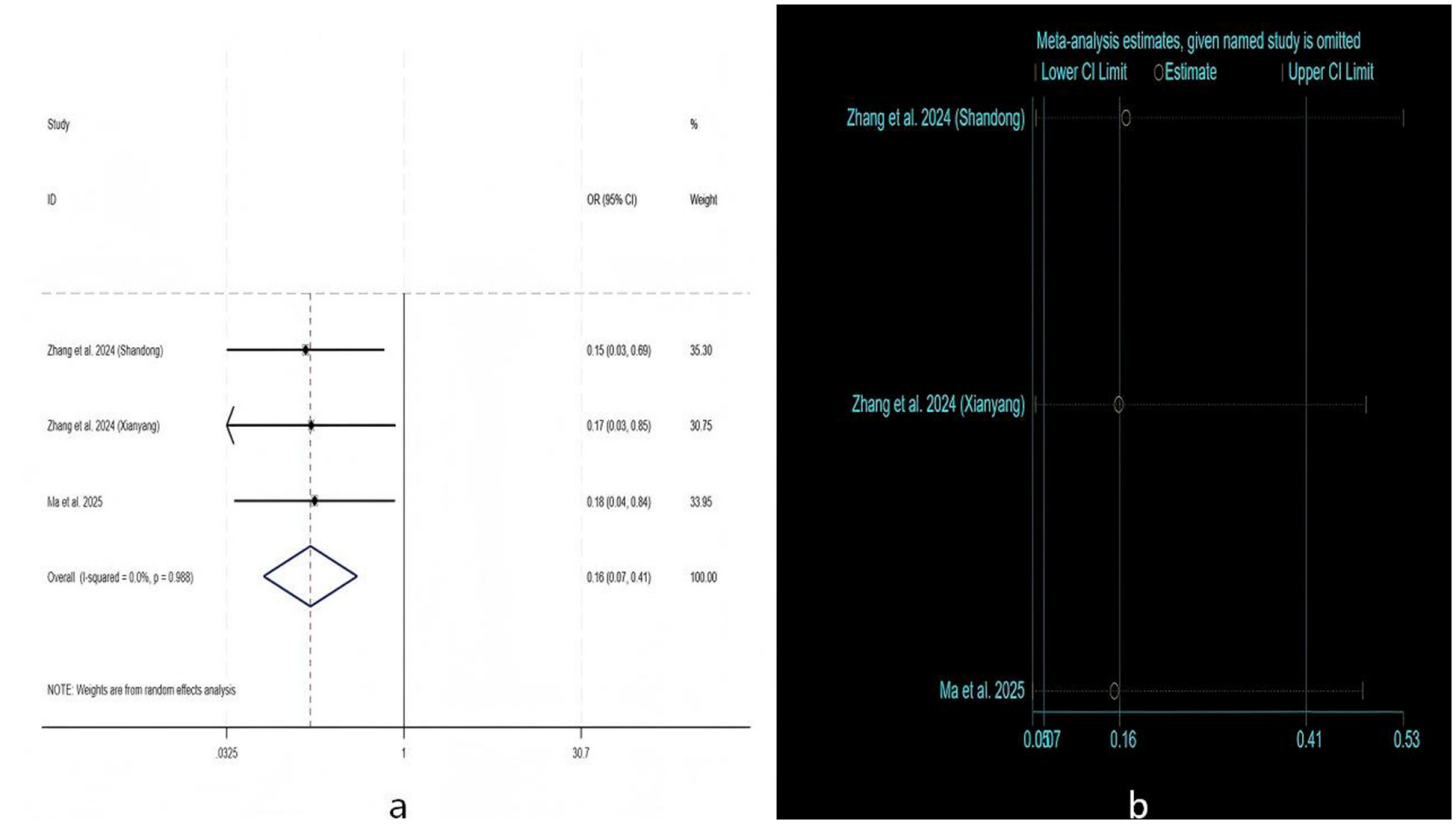
Meta-analysis results for complication rate; **(a)** forest plot; **(b)** sensitivity analysis.

Three studies reported the complication rate and were included in the meta-analysis. A random-effects model was employed for the pooled analysis. The forest plot (Figure 4a) indicated no significant statistical heterogeneity among the studies (I^2^ = 0.0%, *p* = 0.988). The pooled analysis demonstrated that patients receiving airway clearance interventions had a significantly lower risk of complications compared to the control group, with a pooled odds ratio of 0.16 (95% CI: 0.07–0.41). Sensitivity analysis was performed by iteratively removing each individual study (Figure 4b). The results showed that after the removal of any single study, the upper limit of its 95% confidence interval remained below 1, indicating that the core conclusion of a significantly reduced risk of complications from the meta-analysis is robust.

### 3.6 Meta-analysis of arterial oxygen saturation

**Figure 5:**
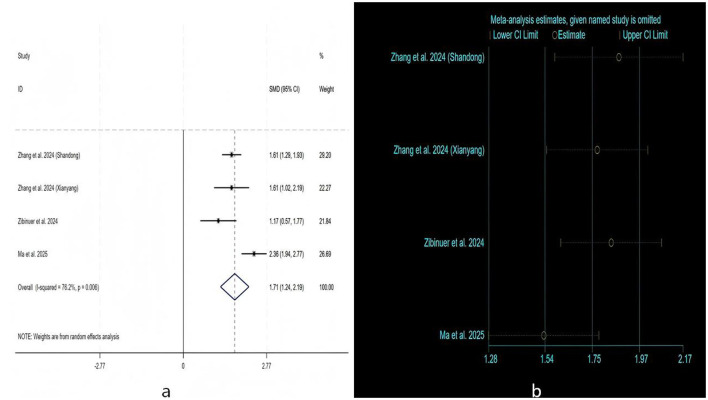
Meta-analysis results for arterial oxygen saturation; **(a)** forest plot; **(b)** sensitivity analysis.

Four studies reported arterial oxygen saturation and were included in the meta-analysis. Baseline and post-intervention SpO2/SaO2 values for each of the four contributing studies are presented in Supplementary Table 5. The standardized mean difference was used as the effect measure for the pooled analysis, employing a random-effects model. The forest plot (Figure 5a) indicated significant statistical heterogeneity among the studies (I^2^ = 76.2%, *p* = 0.006). This considerable heterogeneity may be partly attributed to measurement heterogeneity: three studies reported SaO2 or arterial oxygen saturation measured by blood gas-related assessment, whereas one study reported SpO2 measured by pulse oximetry under oxygen support. Differences in measurement method, indicator type, pneumonia subtype, and baseline severity likely contributed to between-study heterogeneity. The pooled analysis demonstrated a significant improvement in oxygen saturation for patients receiving airway clearance interventions compared to the control group, with a pooled standardized mean difference of 1.71 (95% CI: 1.24–2.19).

To facilitate clinical interpretation, we calculated the between-group net difference attributable to ACTs, rather than within-group pre-post changes. As shown in Supplementary Table 5, the net treatment effect was calculated as the absolute change in the intervention group minus the absolute change in the control group. Zhang et al. (9) (2024, Shandong) showed a net between-group difference of 9.16 percentage points (intervention: +19.96%, control: +10.80%); Zhang et al. (10) (2024, Xianyang) had a net difference of 9.85 percentage points (intervention: +19.95%, control: +10.10%); Zibinuer et al. (12) showed a modest net difference of 2.96 percentage points (intervention: +4.20%, control: +1.24%); and Ma et al. (18) reported a net difference of 11.96 percentage points (intervention: +30.17%, control: +18.21%). Overall, the net clinical benefit of ACTs on oxygen saturation ranged from approximately 3 to 12 percentage points across included studies.

The absolute oxygen saturation change was calculated using post-intervention standard deviations (mean post-intervention SD approximately 4.78%) extracted from Supplementary Table 5, which are the SD values underlying the SMD calculation. The pooled SMD of 1.71 corresponds to an absolute between-group improvement of approximately 8.2 percentage points, reflecting the treatment effect attributable to ACTs after accounting for concurrent improvement in the control groups.

Sensitivity analysis was performed by iteratively removing each individual study (Figure 5b). The results showed that after the removal of any single study, the lower limit of the 95% confidence interval for the pooled SMD remained above zero, indicating that the core conclusion of a positive intervention effect on improving oxygen saturation is robust despite the observed heterogeneity.

### 3.7 Exploratory analysis of the modified medical research council dyspnea score

Three studies reported the modified Medical Research Council Dyspnea Score and were included in an exploratory meta-analysis. It is important to note that the mMRC scale was originally developed and validated for chronic obstructive pulmonary disease; its measurement properties have not been established in acute pneumonia, where respiratory symptoms may fluctuate rapidly and are influenced by fever, systemic inflammation, analgesics, and other acute physiological variables. Therefore, the following results should be interpreted as hypothesis-generating only.

**Figure 6:**
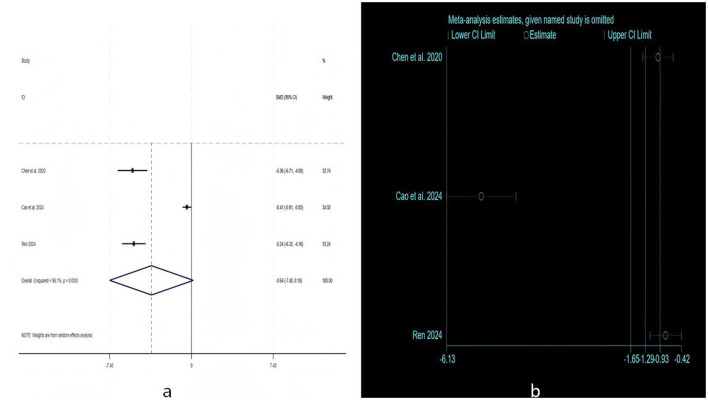
Meta-analysis results for the modified Medical Research Council Dyspnea Score; **(a)** forest plot; **(b)** sensitivity analysis.

The standardized mean difference was used as the effect measure, and a random-effects model was employed for the pooled analysis. The forest plot (Figure 6a) indicated substantial statistical heterogeneity among the studies (I^2^ = 98.1%, *p* < 0.001). The pooled analysis showed a difference in the improvement of the mMRC score between the intervention and control groups, with a pooled standardized mean difference of −3.64 (95% CI: −7.45–0.18). Since the confidence interval crosses the null value (zero), this pooled effect estimate did not reach statistical significance. The sensitivity analysis (Figure 6b) demonstrated that after iteratively removing each study, the point estimate of the pooled effect fluctuated between −6.13 and −1.65, with substantial variation in the range of its 95% confidence interval. This instability, combined with the extreme heterogeneity and the lack of validation of the mMRC scale in acute pneumonia, indicates that the current meta-analytic finding is unreliable and should not be used to draw definitive conclusions about the effect of airway clearance techniques on dyspnea relief in acute pneumonia.

## 4 Discussion

This systematic review synthesized data from 11 recent randomized controlled trials conducted in China, providing the first meta-analysis specifically evaluating the efficacy and safety of airway clearance techniques in adult patients with pneumonia. The primary findings of this study indicate that adjunctive airway clearance techniques, when combined with conventional treatment, can significantly increase the treatment effective rate, significantly reduce the complication rate, and significantly improve arterial oxygen saturation in adult patients with pneumonia. However, due to substantial heterogeneity and wide confidence intervals, we could not draw a definitive conclusion regarding its impact on the modified Medical Research Council Dyspnea Score.

The significant improvement in treatment effective rate (OR = 3.83) carries clear clinical implications, suggesting that airway clearance techniques may enhance the overall effectiveness of conventional therapy by promoting secretion clearance and improving ventilation and oxygenation. This finding aligns with the fundamental physiological goals of respiratory management in critically ill patients (4). The significant reduction in complication rate (approximately a 74% risk reduction) is an important safety signal (19).

Although all included studies reported the interventions as safe and well-tolerated, caution is warranted when generalizing this finding to a more critically ill clinical population, as most studies likely excluded patients with profound hemodynamic instability (20, 21). The significant improvement in oxygen saturation is consistent with pathophysiological expectations (21, 22). The pooled standardized mean difference of 1.71 (95% CI: 1.24–2.19) corresponded to an absolute between-group improvement of approximately 8.2 percentage points when calculated using post-intervention standard deviations. Across individual studies, the net between-group benefit ranged from approximately 3 to 12 percentage points, rather than reflecting the larger within-intervention-arm baseline-to-post changes alone.

Apart from differences in baseline disease severity and pneumonia subtypes, the mixed use of SaO2 and SpO2 across studies is a key source of the substantial heterogeneity (I^2^ = 76.2%) observed for the oxygen saturation outcome. The magnitude of net between-group benefit varied across studies: patients with severe pneumonia requiring mechanical ventilation [Ma et al. (18)] obtained a net gain of nearly 12 percentage points, patients with general adult pneumonia [Zhang et al. (9, 10)] had a net gain of around 9 percentage points, whereas patients with stable severe viral pneumonia [Zibinuer et al. (12)] achieved a modest net gain of approximately 3 percentage points. This variation is closely related to baseline hypoxemia severity, pneumonia type, and measurement method. Despite heterogeneity, all studies demonstrated a positive treatment effect of ACTs on oxygen saturation, and sensitivity analysis confirmed the robustness of this conclusion. Clinicians should consider baseline oxygen saturation, pneumonia type, and the specific oxygenation measurement method when interpreting the expected therapeutic benefit.

The high heterogeneity (I^2^ = 98.1%) and inconclusive result regarding the mMRC score suggest considerable variability in the subjective assessment of dyspnea across studies, potentially related to the timing of assessment, patient education levels, or differences in symptom perception (23–25).

Several limitations of this study should be acknowledged. First, all included studies were conducted in China, which may affect the generalizability of the findings across different healthcare systems and populations. Second, the number of available studies for some outcomes (e.g., complication rate, mMRC score) was limited, reducing the power of the analyses. Third, there was a lack of standardization across studies regarding the specific implementation details of the interventions (e.g., technical parameters, treatment duration) and the timing of outcome assessments, which likely contributed to the heterogeneity observed for some outcomes. Fourth, we acknowledge that treatment effective rate is not a validated core outcome measure in international pneumonia research and that its definition varied in detail across the included Chinese trials. Although all definitions captured a multidimensional clinical response (symptoms, imaging, and laboratory parameters), the composite nature of this endpoint limits comparability with studies using patient-centered outcomes such as mortality or hospital stay. Therefore, the pooled OR of 3.83 should be interpreted as hypothesis-generating and requires confirmation in future trials using standardized, validated endpoints. Fifth, the reliance on studies from lower-visibility Chinese journals raises legitimate concerns regarding publication integrity, including potential outcome reporting bias and data duplication, which are recognized challenges in the systematic review literature. While we assessed each included study using the Cochrane RoB1 tool and found no high-risk domains for most studies, we cannot entirely exclude the possibility of selective reporting or insufficient peer review transparency. Therefore, our pooled estimates should be interpreted with caution when applied to non-Chinese populations or healthcare settings. Sixth, the umbrella term airway clearance techniques in this review encompasses a heterogeneous set of interventions with distinct physiological mechanisms, including positioning, chest wall vibration, nebulization/humidification, fiberoptic bronchoscopy-assisted lavage, high-flow nasal cannula oxygen combined with clearance, and evidence-based refined airway management protocols. These interventions are mechanistically distinct and may not share equivalent efficacy profiles. Ideally, a subgroup analysis by intervention category would be performed to identify which specific modality drives the observed benefit. However, the number of studies within each intervention category was insufficient to conduct meaningful subgroup analyses (e.g., only one study used positioning alone, two used bronchoscopy-assisted clearance, and the remainder used mixed or refined protocols). Therefore, we could not reliably determine which ACT modality contributed most to the positive findings, in which patient subgroup, or through what mechanism. This limitation precludes any confident recommendation of a specific ACT modality and highlights the need for future trials to evaluate standardized, well-defined interventions individually. Seventh, the modified Medical Research Council (mMRC) Dyspnea Scale, used as an outcome measure in three of the included studies, was originally developed and validated for chronic obstructive pulmonary disease (COPD). Its measurement properties have not been established in the context of acute pneumonia. During acute infectious illness, respiratory symptoms can change rapidly over days and are heavily influenced by physiological variables such as fever, systemic inflammation, sepsis, and the use of analgesics or sedatives. These factors introduce substantial variability that is not accounted for by the stable, chronic-disease framework for which the mMRC was designed. Consequently, the extreme heterogeneity (I^2^ = 98.1%) and the non-significant, unstable pooled estimate observed in our exploratory analysis likely reflect, at least in part, inappropriate instrument application rather than true heterogeneity in intervention effects. Therefore, the mMRC findings should be interpreted with great caution and are presented only as hypothesis-generating. Future studies should use validated dyspnea assessment tools specifically adapted for acute pneumonia or should consider alternative patient-reported outcome measures that are sensitive to rapid clinical changes. Eighth, the risk of bias assessment revealed that several studies had unclear risk for allocation concealment and other bias (e.g., baseline imbalance, early stopping). Unclear allocation concealment may introduce selection bias, while unclear other bias could affect the reliability of effect estimates. Although these uncertainties exist, sensitivity analyses for the primary outcomes (treatment effective rate, complication rate) showed robust results, and the GRADE certainty was downgraded accordingly. Readers should interpret the findings with these potential biases in mind. Future international multicenter trials are urgently needed to confirm the external validity of these findings. Finally, this study did not evaluate longer-term patient-important outcomes such as rehospitalization rates, quality of life, or mortality. This meta-analysis was retrospectively registered, which may partially affect the objectivity of the results.

From a clinical practice perspective, the evidence from this study is hypothesis-generating and suggests that airway clearance techniques may offer short-term benefits in treatment effectiveness, complication reduction, and oxygenation improvement for hospitalized adult pneumonia patients. However, given the heterogeneity of interventions, the use of a non-validated composite primary outcome, the geographic restriction to Chinese trials, and the absence of long-term patient-centered outcomes, these findings should not be interpreted as definitive practice-changing evidence. Any clinical consideration of airway clearance techniques should be weighed against these limitations, and corroboration from international multicenter trials using standardized intervention protocols and validated outcome measures is urgently needed before routine implementation can be recommended.

In conclusion, this systematic review and meta-analysis provides hypothesis-generating evidence that adjunctive airway clearance techniques, when combined with conventional treatment, may increase treatment effectiveness, reduce complication risk, and improve arterial oxygen saturation in adult patients with pneumonia. However, due to substantial heterogeneity, methodological limitations (including the use of a non-validated composite outcome and lack of prospective protocol registration), and the absence of long-term patient-centered outcomes, these findings require confirmation in future international multicenter trials with standardized interventions and validated endpoints. Currently, no definitive practice recommendation can be made, and clinicians should interpret the results with caution.

## Data Availability

The original contributions presented in the study are included in the article/Supplementary material, further inquiries can be directed to the corresponding author.
