# Supplementary material for: Efficacy and safety of airway clearance techniques in Chinese adult patients with pneumonia: a systematic review and meta-analysis
Source: Front Med (Lausanne). 2026 Jul 22;13:1819629. doi: 10.3389/fmed.2026.1819629 (PMC13437794; doi:10.3389/fmed.2026.1819629)
Supplement: Supplementary file 2 [file Table_2.docx]

Supplementary Table 2 Search Strategy

Pubmed

#1 ("Pneumonia"[Mesh] OR "Pneumonia, Ventilator-Associated"[Mesh] OR "Pneumonia, Aspiration"[Mesh] OR "Community-Acquired Infections"[Mesh] OR pneumon*[tiab] OR (respiratory[tiab] AND (infection*[tiab] OR inflam*[tiab])) OR "chest infection*"[tiab])

#2 ("Adult"[Mesh] OR adult*[tiab] OR aged[tiab] OR elderly[tiab] OR "middle aged"[tiab] OR "older patient*"[tiab])

#3 #1 AND #2

#4 ("Airway Clearance Techniques"[Mesh] OR "Respiratory Therapy"[Mesh] OR "Chest Physiotherapy"[tiab] OR "Airway Clearance"[tiab] OR "Active Cycle of Breathing Technique*"[tiab] OR "ACBT"[tiab] OR "Autogenic Drainage"[tiab] OR "Forced Expiration Technique*"[tiab] OR "Percussion"[Mesh] OR "Postural Drainage"[Mesh] OR "High-Frequency Chest Wall Compression"[tiab] OR "HFCWO"[tiab] OR "Mechanical Insufflation-Exsufflation"[tiab] OR "Cough Assist"[tiab] OR "Manual Hyperinflation"[tiab] OR "vibration*"[tiab] OR "mucus clearance"[tiab] OR "sputum clearance"[tiab] OR "bronchial hygiene"[tiab])

#5 ("Randomized Controlled Trial" [Publication Type] OR "Randomized Controlled Trials as Topic"[Mesh] OR "controlled clinical trial"[pt] OR random*[tiab] OR trial[tiab] OR groups[tiab])

#6 #3 AND #4 AND #5

Web of Science

#1 TS=(pneumon* OR “community-acquired pneumonia” OR “ventilator-associated pneumonia” OR “aspiration pneumonia” OR “respiratory tract infection” OR “chest infection” OR “lung inflammation”)

#2 TS=(adult* OR aged OR elderly OR “middle aged” OR “older patient*” OR “older adult*”)

#3 #1 AND #2

#4 TS=(“airway clearance” OR “airway clearance technique*” OR “chest physiotherapy” OR “respiratory therapy” OR “active cycle of breathing” OR “ACBT” OR “autogenic drainage” OR “forced expiration technique*” OR “postural drainage” OR “percussion” OR “high frequency chest wall compression” OR “HFCWO” OR “mechanical insufflation-exsufflation” OR “cough assist” OR “manual hyperinflation” OR “mucus clearance” OR “sputum clearance” OR “bronchial hygiene” OR “vibration*”)

#5 TS=(random* OR trial OR “controlled trial” OR “clinical trial” OR groups)

#6 #3 AND #4 AND #5

Cochrane Library

#1 MeSH descriptor: [Pneumonia] explode all trees

#2 MeSH descriptor: [Pneumonia, Ventilator-Associated] this term only

#3 MeSH descriptor: [Pneumonia, Aspiration] this term only

#4 MeSH descriptor: [Community-Acquired Infections] this term only

#5 (pneumon* or “chest infection*” or “respiratory infection*” or “lung inflammation”):ti,ab,kw

#6 #1 or #2 or #3 or #4 or #5

#7 MeSH descriptor: [Adult] this term only

#8 (adult* or aged or elderly or “middle aged” or “older patient*”):ti,ab,kw

#9 #7 or #8

#10 #6 and #9

#11 MeSH descriptor: [Airway Clearance Techniques] this term only

#12 MeSH descriptor: [Respiratory Therapy] this term only

#13 MeSH descriptor: [Percussion] this term only

#14 MeSH descriptor: [Postural Drainage] this term only

#15 (“airway clearance” or “chest physiotherapy” or “active cycle of breathing” or ACBT or “autogenic drainage” or “forced expiration technique*” or “postural drainage” or percussion or “high frequency chest wall oscillation” or HFCWO or “mechanical insufflation-exsufflation” or “cough assist” or “manual hyperinflation” or “mucus clearance” or “sputum clearance” or “bronchial hygiene” or vibrat*):ti,ab,kw

#16 #11 or #12 or #13 or #14 or #15

#17 #10 and #16

CNKI

(SU=('pneumonia' OR 'community-acquired pneumonia' OR 'hospital-acquired pneumonia' OR 'ventilator-associated pneumonia' OR 'aspiration pneumonia' OR 'bacterial pneumonia' OR 'viral pneumonia' OR 'severe pneumonia') AND SU%=('adult' OR 'aged' OR 'elderly' OR 'middle-aged' OR 'geriatric') AND SU%=('airway clearance' OR 'airway clearance technique' OR 'chest physiotherapy' OR 'respiratory therapy' OR 'active cycle of breathing technique' OR 'ACBT' OR 'autogenic drainage' OR 'forced expiration technique' OR 'postural drainage' OR 'percussion' OR 'vibration' OR 'manual hyperinflation' OR 'mechanical insufflation-exsufflation' OR 'high-frequency chest wall oscillation' OR 'HFCWO' OR 'sputum clearance' OR 'mucus clearance' OR 'bronchial hygiene' OR 'cough assist')) AND (FT=' Randomized Controlled Trial ' OR FT='RCT' OR FT='controlled clinical trial')

Wanfang

(SU=('pneumonia' OR 'community-acquired pneumonia' OR 'hospital-acquired pneumonia' OR 'ventilator-associated pneumonia' OR 'aspiration pneumonia' OR 'bacterial pneumonia' OR 'viral pneumonia' OR 'severe pneumonia') AND SU%=('adult' OR 'aged' OR 'elderly' OR 'middle-aged' OR 'geriatric') AND SU%=('airway clearance' OR 'airway clearance technique' OR 'chest physiotherapy' OR 'respiratory therapy' OR 'active cycle of breathing technique' OR 'ACBT' OR 'autogenic drainage' OR 'forced expiration technique' OR 'postural drainage' OR 'percussion' OR 'vibration' OR 'manual hyperinflation' OR 'mechanical insufflation-exsufflation' OR 'high-frequency chest wall oscillation' OR 'HFCWO' OR 'sputum clearance' OR 'mucus clearance' OR 'bronchial hygiene' OR 'cough assist')) AND (FT=' Randomized Controlled Trial ' OR FT='RCT' OR FT='controlled clinical trial')
