# Supplementary material for: Efficacy and safety of airway clearance techniques in Chinese adult patients with pneumonia: a systematic review and meta-analysis
Source: Front Med (Lausanne). 2026 Jul 22;13:1819629. doi: 10.3389/fmed.2026.1819629 (PMC13437794; doi:10.3389/fmed.2026.1819629)
Supplement: Supplementary file 3 [file Table_3.docx]

Supplementary Table 3. Operational Definitions of “Treatment Effective Rate” in the Seven Included Studies

| Included Study | English Translation | Assessment Criteria |
| --- | --- | --- |
| Cao et al. 2024 | Curative effect criteria: Markedly effective: clinical symptoms and signs (cough, sputum production, dyspnea) essentially disappeared, pulmonary moist rales essentially resolved, chest X-ray showed pulmonary infiltrates essentially resolved or markedly reduced, laboratory parameters (WBC, neutrophil percentage, CRP, etc.) returned to normal or significantly improved. Effective: the above clinical symptoms and signs improved, pulmonary moist rales decreased, chest X-ray showed some resolution of pulmonary infiltrates, laboratory parameters improved. Ineffective: no significant improvement or worsening of any of the above indicators. Effective rate = (markedly effective + effective) / total cases × 100%. | Multidimensional clinical response: symptoms, physical signs, chest imaging, and laboratory parameters. |
| Mo et al. 2023 | Curative effect criteria: Markedly effective: body temperature returned to normal, clinical symptoms and signs (cough, sputum production, dyspnea, etc.) essentially resolved, peripheral WBC and neutrophil percentage returned to normal, chest X-ray showed essentially complete resolution of pulmonary infiltrates. Effective: body temperature normalized or markedly decreased, clinical symptoms and signs improved, peripheral WBC and neutrophil percentage improved, chest X-ray showed partial resolution of pulmonary infiltrates. Ineffective: no improvement or worsening of any of the above indicators. Effective rate = (markedly effective + effective) / total cases × 100%. | Multidimensional clinical response: body temperature, symptoms, laboratory parameters, and chest imaging. |
| Ren 2024 | Curative effect criteria: Markedly effective: cough, sputum production, dyspnea completely resolved, pulmonary rales resolved, SpO₂ returned to normal (≥95%), chest CT showed essentially complete resolution of pulmonary lesions. Effective: clinical symptoms markedly reduced, pulmonary rales decreased, SpO₂ improved (≥5% increase from baseline), chest CT showed partial resolution of pulmonary lesions. Ineffective: no improvement or worsening of any of the above indicators. Effective rate = (markedly effective + effective) / total cases × 100%. | Multidimensional clinical response: symptoms, physical signs, SpO₂, and chest imaging. |
| Lu et al. 2025 | Curative effect criteria: Markedly effective: clinical symptoms (fever, cough, sputum production, dyspnea, etc.) essentially resolved, blood gas parameters (PaO₂, PaCO₂, PaO₂/FiO₂) significantly improved, chest imaging showed essentially complete resolution of pulmonary infiltrates, duration of mechanical ventilation significantly reduced. Effective: clinical symptoms reduced, blood gas parameters improved, chest imaging showed partial resolution of pulmonary infiltrates, duration of mechanical ventilation reduced. Ineffective: no improvement or worsening of any of the above indicators. Effective rate = (markedly effective + effective) / total cases × 100%. | Multidimensional clinical response: symptoms, blood gas parameters, chest imaging, and mechanical ventilation duration. |
| Lin et al. 2024 | Curative effect criteria: Markedly effective: clinical symptoms (fever, cough, sputum production, etc.) resolved, clear breath sounds on auscultation, SpO₂ returned to normal (≥95%), chest CT showed essentially complete resolution of pulmonary lesions, no complications. Effective: clinical symptoms markedly reduced, breath sounds improved, SpO₂ improved (≥3% increase from baseline), chest CT showed partial resolution of pulmonary lesions, reduced complication rate. Ineffective: no improvement or worsening of any of the above indicators. Effective rate = (markedly effective + effective) / total cases × 100%. | Multidimensional clinical response: symptoms, physical signs, SpO₂, chest imaging, and complications. |
| Ma et al. 2025 | Curative effect criteria: Markedly effective: clinical symptoms (fever, cough, sputum production, dyspnea) completely resolved, pulmonary rales resolved, blood gas parameters (PaO₂, PaO₂/FiO₂) returned to normal, chest CT showed essentially complete resolution of pulmonary infiltrates. Effective: clinical symptoms markedly reduced, pulmonary rales decreased, blood gas parameters improved (PaO₂ increased by ≥10 mmHg), chest CT showed partial resolution of pulmonary infiltrates. Ineffective: no improvement or worsening of any of the above indicators. Effective rate = (markedly effective + effective) / total cases × 100%. | Multidimensional clinical response: symptoms, physical signs, blood gas parameters, and chest imaging. |
| Wang et al. 2025 | Curative effect criteria: Markedly effective: cough and sputum production essentially resolved, sputum viscosity changed from thick to thin, sputum volume significantly reduced, SpO₂ returned to normal (≥95%), chest X-ray showed essentially complete resolution of pulmonary opacities. Effective: clinical symptoms reduced, sputum viscosity decreased, sputum volume reduced, SpO₂ improved (≥3% increase from baseline), chest X-ray showed partial resolution of pulmonary opacities. Ineffective: no improvement or worsening of any of the above indicators. Effective rate = (markedly effective + effective) / total cases × 100%. | Multidimensional clinical response: symptoms, sputum characteristics, SpO₂, and chest imaging. |
