# Supplementary material for: Efficacy and safety of airway clearance techniques in Chinese adult patients with pneumonia: a systematic review and meta-analysis
Source: Front Med (Lausanne). 2026 Jul 22;13:1819629. doi: 10.3389/fmed.2026.1819629 (PMC13437794; doi:10.3389/fmed.2026.1819629)
Supplement: Supplementary file 4 [file Table_4.docx]

Supplementary Table 4. GRADE Evidence Certainty Assessment

| Outcome | Number of studies | Risk of bias | Inconsistency | Indirectness | Imprecision | Publication bias | Overall certainty of evidence | Reason for downgrading |
| --- | --- | --- | --- | --- | --- | --- | --- | --- |
| Treatment effective rate | 7 | Not serious | Not serious | Serious | Not serious | None | ⊕⊕⊕◯ Moderate | Composite endpoint not validated in international pneumonia research; definitions varied slightly across studies |
| Complication rate | 3 | Serious | Not serious | Not serious | Serious | None | ⊕⊕◯◯ Low | Unclear allocation concealment in some studies; Small sample size (total <400) |
| Arterial oxygen saturation | 4 | Not serious | Serious | Serious | Not serious | None | ⊕⊕◯◯ Low | Considerable heterogeneity (I^2^=76.2%); Indirectness of outcome measure (surrogate) |
| mMRC Dyspnea Score | 3 | Serious | Serious | Serious | Serious | None | ⊕⊕◯◯ Low | Performance bias (some concerns); Extreme heterogeneity (I^2^=98.1%); mMRC not validated for acute pneumonia; ³Small sample size |
