# Supplementary material for: Efficacy and safety of airway clearance techniques in Chinese adult patients with pneumonia: a systematic review and meta-analysis
Source: Front Med (Lausanne). 2026 Jul 22;13:1819629. doi: 10.3389/fmed.2026.1819629 (PMC13437794; doi:10.3389/fmed.2026.1819629)
Supplement: Supplementary file 5 [file Table_5.docx]

Supplementary Table 5. Baseline and Post-Intervention SpO2/SaO2 Values and Net Between-Group Differences

| **Study** | **Group** | **Baseline (%)** | **Post-intervention (%)** | **Absolute change (%)** | **Notes** | **Net between-group difference (%)** |
| --- | --- | --- | --- | --- | --- | --- |
| Zhang et al. 2024 (Shandong) | Intervention (n=100) | 75.89 +/- 3.39 | 95.85 +/- 6.22 | +19.96 | SaO2, blood gas analyzer | 9.16 |
| Zhang et al. 2024 (Shandong) | Control (n=100) | 75.71 +/- 3.44 | 86.51 +/- 5.35 | +10.80 | SaO2, blood gas analyzer |  |
| Zhang et al. 2024 (Xianyang) | Intervention (n=30) | 75.91 +/- 3.37 | 95.86 +/- 6.23 | +19.95 | SaO2, blood gas analyzer | 9.85 |
| Zhang et al. 2024 (Xianyang) | Control (n=30) | 76.43 +/- 3.42 | 86.53 +/- 5.34 | +10.10 | SaO2, blood gas analyzer |  |
| Zibinuer et al. 2024 | Intervention (n=25) | 90.56 +/- 1.94 | 94.76 +/- 2.88 (day 21) | +4.20 | SpO2 under oxygen support | 2.96 |
| Zibinuer et al. 2024 | Control (n=25) | 90.72 +/- 2.11 | 91.96 +/- 1.79 (day 21) | +1.24 | SpO2 under oxygen support |  |
| Ma et al. 2025 | Intervention (n=75) | 59.85 +/- 5.43 | 90.02 +/- 5.08 | +30.17 | SO2/arterial oxygen saturation in mechanically ventilated severe pneumonia | 11.96 |
| Ma et al. 2025 | Control (n=75) | 59.81 +/- 5.51 | 78.02 +/- 5.11 | +18.21 | SO2/arterial oxygen saturation in mechanically ventilated severe pneumonia |  |

Notes:

Data are presented as mean +/- standard deviation. Absolute change = post-intervention value minus baseline value. Net between-group difference = intervention-group absolute change minus control-group absolute change. Mean post-intervention SD across contributing groups was approximately 4.78%, which was used for SMD back-conversion.

Zhang et al. 2024 (Shandong) and Zhang et al. 2024 (Xianyang) used SaO₂ (arterial oxygen saturation) measured by blood gas analyzer.

Zibinuer et al. 2024 reported SpO₂ (pulse oxygen saturation) under oxygen support; the post‑intervention measurement was taken at day 21.

Ma et al. 2025 reported SO₂ in mechanically ventilated patients with severe pneumonia; the low baseline values reflect the severity of illness.
